# From expression pattern to genetic association in asthma and asthma-related phenotypes

**DOI:** 10.1186/1756-0500-5-630

**Published:** 2012-11-13

**Authors:** Vanessa T Vaillancourt, Martine Bordeleau, Michel Laviolette, Catherine Laprise

**Affiliations:** 1 Département des sciences fondamentales, Université du Québec à Chicoutimi, 555 boulevard de l’Université, Chicoutimi, Saguenay, Québec, G7H 2B1, Canada; 2 Institut de cardiologie et de pneumologie de l’Hôpital Laval, Université Laval, 2725, chemin Sainte-Foy, Québec, G1V 4G5, Canada

**Keywords:** Asthma, Atopy, Atopic asthma, Microarrays, Association study, Expression profile, *AGC1*, *ADRA2A*, *IFI6*, *ADH1B*

## Abstract

**Background:**

Asthma is a complex disease characterized by hyperresponsiveness, obstruction and inflammation of the airways. To date, several studies using different approaches as candidate genes approach, genome wide association studies, linkage analysis and genomic expression leaded to the identification of over 300 genes involved in asthma pathophysiology. Combining results from two studies of genomic expression, this study aims to perform an association analysis between genes differently expressed in bronchial biopsies of asthmatics compared to controls and asthma-related phenotypes using the same French-Canadian Caucasian population.

**Results:**

Before correction, 31 of the 85 genes selected were associated with at least one asthma-related phenotype. We found four genes that survived the correction for multiple testing. The rs11630178 in aggrecan gene (*AGC1)* is associated with atopy (p=0.0003) and atopic asthma (p=0.0001), the rs1247653 in the interferon alpha-inducible protein 6 *(IFI6)*, the rs1119529 in adrenergic, alpha-2A-, receptor (*ADRA2A)* and the rs13103321 in the alcohol dehydrogenase 1B (class I), beta polypeptide (*ADH1B),* are associated with asthma (p=0.019; 0.01 and 0.002 respectively).

**Conclusion:**

To our knowledge, this is the first time those genes are associated with asthma and related traits. Consequently, our study confirms that genetic and expression studies are complementary to identify new candidate genes and to investigate their role to improve the comprehension of the complexity of asthma pathophysiology.

## Background

Asthma is a complex disease which is characterized by hyperresponsiveness, obstruction and inflammation of the airways and is modulated by the interaction of genetic and environmental factors [1]. The appearance of an immune response and of a remodeling process, which implicates structural and inflammatory cells, dictates the development, the severity and the chronicity of asthma [2]. Atopy, which also shows a genetic component, is characterized by the excessive production of immunoglobulin E in response to environmental allergens and is an important risk factor of asthma [3]. Using candidate genes approach, genome wide association studies, linkage analysis or genomic expression, studies of the last decades revealed that the pathophysiology of asthma is related with more than 300 genes [4,5]. Indeed, several studies showed that microarrays are a relevant tool to highlight genes that are implicated in the different processes occurring during asthma. [6,7] This study aims firstly to demonstrate that expression and genetic association studies complement each other and secondly to test the hypothesis that differences in the expression of several genes identified in the studies of Laprise *et al.*[8] and Chamberland *et al.*[9] could be caused by genetic variants. To achieve this, we combined the results of Laprise *et al*. and Chamberland *et al*. and performed association analysis between the differently expressed genes and asthma-related phenotypes in the family sample of Saguenay–Lac-St-Jean (SLSJ). The present study identified four significantly associated genes, which are the aggrecan gene (*AGC1)* the interferon alpha-inducible protein 6 *(IFI6)*, the adrenergic, alpha-2A-, receptor (*ADRA2A)* and the alcohol dehydrogenase 1B (class I), beta polypeptide (*ADH1B)*.

## Methods

### Selection of genes

The selection of genes was made according to the results of two previous studies of Laprise *et al.* and Chamberland *et al*. [8,9]. Both studies included bronchial biopsies from four asthmatic subjects and four controls (from the Respiratory Health Network biobank of the Fonds de la recherche du Québec en santé (FRQS)) and aimed to identify differently expressed genes related to asthma. The study of Laprise *et al*. (Series GSE15823 on GEO) used the U95Av2 array and a t-test with Bonferroni correction with a fold change threshold of 2.0, whereas Chamberland *et al.* (Series GSE41649 on GEO**)** used the U133A array and Smyth’s moderated t-test with a fold change threshold of 1.8 as selection criteria. Both of them had a signification level at 0.05. Those two studies allowed to identify a total of 100 differently expressed genes and classified them in 13 functional groups (see Table 1).

**Table 1:** Differently expressed genes in two microarrays studies using bronchial tissues of asthmatic and control subjects

**Functional category**	**Genes**
Immune signaling molecules	CX3CR1*^†^*, IL7R*^†^, CD14*^†^*, SFRP1*^†^, SCYA21, SDF1, TNFRSF7, TRA@, CD19, ALOX15*^†^*, NOS2A*^†^*, SCYA5, IL2RB*^†^, IL1R2*^†^
Extracellular proteins	*COL11A1, FMOD, MUC5AC*^*£*^*, MUC2*^*£*^*, COL2A1, AGC1, FBLN1, GPC3, MUC4*^*£*^*, SFRP4*
Immune response	IGKC, HLA-DPB1*, HLA-DQA1*, HLA-DQB1*, HLA-DRB4, TRBA^*£*^, MS4A1, IGHM, IGLJ3, CEACAM5, ERAP2, IGHG1*, IGJ, IGLA, IGKV1D-13
Intracellular signaling	*PTPRC,IFI6, ARHGEF16, PSPHL, GNB2, SYK*^*£*^*, PPP1R3C, PHLDA1, TYROBP, FKBP8, PIM2, FLRT3*
Proteolytic enzymes	CPA3*, GZMA, CTSC, CTSG^*£*^*, PSMA6, TPSB1*^*£*^, SERPINB2, SERPINB4*, SPINK5
Transmembrane proteins	*TM4SF3, KLRC3, LGALS7, NKG7, ARHGAP4, ADRA2A, CLCA2, CLDN10, KCNJ16*
Free radical metabolism	GPX3, GSTA2, CYBA*, CYBB, NQO1*^†^
Gene transcription	*TCF21, ZNF38, LDB1, MAF, STAT5A*
Cell growth and proliferation	*GAS1, IGFBP6, CORO1A, TRIM16, EMP3, OGN, S100A14, S100P, SFN*
Cell adhesion molecules	ITGB2, POSTN*
Complement components	*C1QB, C7*^*£*^*, CFD*
Metabolic enzymes	*RBP4, ALDH3A1, FA2H, ADH1B, PIK3R1*
Structural proteins	*KRT23, TNC*^*£*^

* Genes associated with asthma, atopy or atopic asthma.

^†^ Association between these genes and asthma or asthma-related phenotype (atopy or allergic asthma) was already investigated in other articles using the SLSJ familial asthma study [10-12,45].

^£^ Protein related to asthma.

For the present study, some of the 100 genes were not considered for the following reasons: 1) in the first functional group named immune signaling molecules (Table 1), 9 genes (*CX3CR1*, *IL7R, CD14, SFRP1, ALOX15, NOS2A, IL2RB, NQO1* and *IL1R2*) were already included in previous studies and consequently removed for the present study [10-12]; 2) no SNP for the *SDF1, COROA1* and *SFN* genes were available in the Illumina 610KQuad array (genome wide data used for the present study); 3) the TRA@ and TRB@ regions were not included in the present study as they identify loci and contained several genes and 4) no reference sequence was available for the *IGHM* gene. Consequently, a total of 85 genes were included in this genetic association study.

### Sample

The sample comprises 253 asthmatic probands and their family members (a total of 1284 subjects) from SLSJ, a region of the province of Quebec, Canada (see Table 2). This French-Canadian population is known for its founder effect and has wide conserved chromosomal regions, which can facilitate the identification of genes associated with complex traits [13]. In this family collection, 47% present asthma, 53% present atopy, and 34% have asthma and atopy and this sample has been well described in previous publications [14,15]. Phenotypes were defined following the American Thoracic Society’s criteria [16]. Asthma was defined if the subject had an asthma diagnosis by a physician or if he showed a positive methacholine provocation test (PC_20_ > 8 mg/ml) combined with asthma-related symptoms. Atopy was defined as a positive response (wheal diameter ≥ 3 mm at 10 min) for at least one allergen to the skin prick test. Atopic asthma was defined as a combination of both asthma and atopy. This research project was reviewed and approved by the institutional ethic committee of the Université du Québec à Chicoutimi. The written informed consent for participation in the study was obtained from participants or, where participants are children, from a parent.

**Table 2:** Clinical characteristics of the French-Canadian family study

	**Probands**^**a**^	**Family members**
	**n = 253**	**n = 1031**
Male: Female ratio	1 : 1.1	1 : 1.2
Mean age, yr (range)	18 (3–50)	44 (2–93)
Smoking status, n (%)		
Never	211 (85)	473 (47)
Ex-smoker	12 (5)	342 (34)
Smoker	25 (10)	197 (19)
FEV_1_, % predicted (SD) ^b^	92.7 (15.7)	94.6 (20.3)
PC_20_, mg/ml (SD) ^c^	2.53 (3.51)	10.06 (4.97)
Serum IgE in μg/l (SD) ^d^	254.2 (4.9)	112.4 (4.4)
Number of persons with subphenotypes (%)		
Asthma ^e^	253 (100)	378 (37)
Atopy ^f^	200 (79)	509 (49)
Atopic asthma (Asthma + Atopy)	200 (79)	257 (25)

^a^ Probands are first affected family member recruited in the familial collection and family members refers to other family members who joined the study.

^b^ FEV_1_ = Mean calculated for the forced expiratory volume in one second evaluated for 217 probands and 770 family members.

^c^ PC_20_ = Provocative concentration of methacholine that induces a 20% fall in FEV_1_. Geometric mean and SD evaluated for 192 probands and 711 family members.

^d^ IgE = Immunoglobulin E serum concentration. Geometric mean and SD evaluated for 237 probands and 820 family members.

^e^ Present asthma or past documented clinical history of asthma. The reported mean age of onset is 7 years among the probands and 22 years among the asthmatic family members. Asthma phenotype was evaluated for 1022 subjects.

^f^ Defined as at least one positive response on skin prick test (wheal diameter > 3 mm at 10 minutes). Atopy phenotype was evaluated for 997 subjects.

### Genotyping

The sample used in the present study was genotyped using Illumina 610KQuad array (Illumina, San Diego, USA) at the Centre National de Génotypage (CNG, Evry, France) [17]. A total of 1217 SNPs that are located in a ± 10 kb area from the 85 selected genes and fulfilling the following quality control criteria were considered in the analyses: call rate ≥ 95%, minor allele frequency ≥ 5%, Hardy-Weinberg equilibrium p value ≥ 5% and Mendelian errors ≤ 1%. Genotypes were extracted from the genome wide association study (GWAS) data using PLINK software ( http://pngu.mgh.harvard.edu/∼purcell/plink/, version 1.07).

### Statistical analyses

To identify associations between SNPs and asthma-related phenotypes, transmission disequilibrium was analyzed using the Family-Based Association Tests software (FBAT) (version 2.0.3, http://www.biostat.harvard.edu/∼fbat/default.thml). SNP associations were investigated under an additive genetic model and with the empirical variance estimator “-e” to account for the inclusion of multiple affected family members [18]. All the associations of SNPs obtained had to survive a multiple test correction for the number of independent tagSNPs [19] and the number of independent phenotypes, which was identified as two using a matrix spectral decomposition (matSpD) ( http://gump.qimr.edu.au/general/daleN/matSpD/).

## Results

For the analyses, a total of 1217 SNPs located in the 85 studied genes were tested individually for association with asthma, atopy, and atopic asthma. Before correction, 95 SNPs located in 30 genes (*CD19COL11A1*, *MUC2*, *COL2A1*, *AGC1*, *FBLN*, *HLA-DPB1*, *MS4A1*, *CEACAM5*, *IGHG1*, *IGLA*, *PTPRC*, *IFI6*, *SYK*, *CTSC*, *PSMA6*, *SPINK5*, *KLRC3*, *ADRA2A*, *CLCA2*, *KCNJ16*, *CYBA*, ZNF38, *ITGB2*, *C7*, *ALDH3A1*, *FA2H*, *ADH1B*, *PIK3R1* and *TNC)* were associated with asthma-related phenotypes (data not shown, see Additional file 1).

After correction for independent tagSNPs and phenotypes, four SNPs in the *AGC1*, *IFI6*, *ADRA2A* and *ADH1B* genes remained associated (see Table 3). The rs11630178 in the *AGC1* gene is associated with atopy (p=0.0003) and atopic asthma (p=0.0001), the rs1247653 located in the *IFI6* gene (p=0.019), the rs1119529 in the *ADRA2A* gene (p=0.009) and the rs13103321 in the *ADH1B* gene (p=0.002), are associated with asthma.

**Table 3:** Associated SNPs after correction with asthma-related phenotypes

**Gene/ Corr. threshold**^**a**^	**SNP**	**Allele MAF**^**b**^	**Asthma**			**Atopy**			**Atopic asthma**		
			**Fam**^**c**^	**Z**	**P value**	**Fam**	**Z**	**P value**	**Fam**	**Z**	**P value**
***AGC1*** /0.001	rs11630178	T/C 0.34	99	2.69	0.0070	82	3.59	**0.0003**	85	3.90	**0.0001**
***IFI6*** /0.025	rs11247653	A/G 0.43	133	−2.35	**0.0186**	109	−1.77	0.0770	110	−1.90	0.0570
***ADRA2A*** /0.025	rs11195299	G/A 0.06	56	−2.59	**0.0097**	54	−0.79	0.4283	53	−1.92	0.0527
***ADH1B*** /0.003	rs13103321	G/T 0.48	135	−3.04	**0.0024**	114	−2.57	0.0103	114	−2.62	0.0088

^a^ Corrected threshold for multiple testing (considering independent tagSNPs and phenotypes).

^b^ Minor allele frequency observed in the sample.

^c^ Number of families that contributed to the test.

SNP= Single nucleotide polymorphism; MAF= Minor allele frequency; Fam= Families.

## Discussion

This study aimed to demonstrate that gene expression studies may lead to the identification of genetic determinants. Indeed, although all expression differences cannot be explained by a genetic variant, it is possible that differential expression is related to a SNP in a regulatory region or in the promoter [20]. As the genotypes for the SLSJ family study were available from the GWAS data, we investigated the association of SNPs located in genes that are differently expressed in bronchial biopsies of atopic asthmatics compared to controls from two previous publications and asthma-related phenotypes [8,9]. We showed that four genes are genetically associated with asthma or asthma-related phenotypes (the *AGC1* gene associated with atopy and atopic asthma, the *IFI6*, *ADRA2A* and *ADH1B* genes associated with asthma).

Like the majority of asthma associated SNPs, the variants associated in the present study are located in introns [20]. Although, mutations located in an intron may lead to the suppression of critical steps (such as the binding of splicing factors or DNA polymerase) and to the abolition or creation of a binding site causing alteration in gene expression [21].

The *AGC1* gene, located in the chromosomal region 15q26, is coding for the aggrecan, a major component of the extracellular matrix of the cartilage that binds with tenascins [22]. More specifically, it binds to the type C tenascins that are upregulated in acute and chronic inflammatory diseases [23]. In asthmatic subjects, there is an abnormal deposition of tenascin C on the basal membrane, which leads to a decrease of the airway lumen [24]. Tenascin C is implicated in allergies and increases during seasonal atopic asthma [25]. Three SNPs in the tenascin C gene, including a coding SNP (GLN/1978/ARG), were associated with atopy in our study before correction (data not shown). The implication of the tenascin C in the atopic mechanisms supports its association with atopy and atopic asthma phenotypes in our study, but this gene was also associated with asthma in other studies [26]. In the literature, the *AGC1* gene was mostly associated with skeleton and spinal cord diseases [27,28].

The *IFI6* gene is located on the chromosome 1 and codes for an interferon-induced protein that regulates the apoptosis process. This gene is involved in the treatment of myeloma and other malignancy diseases by its antiapoptotic activity [29]. There is no function established for this gene in respiratory disorder except for its implication in tract infection caused by the respiratory syncytial virus (RSV) [30]. As RSV leads to the inflammation of the epithelium in affected patients and as a previous GWAS demonstrated that genes implicated in the epithelial function are strongly associated with asthma [17], the *IFI6* gene could play a role in asthma through the modulation of the epithelium structure.

The *ADRA2A* gene, mapped on the 10q23 chromosomal region, codes for the A subtype of the adrenergic g protein-coupled receptors family. This family plays a role in the regulation of neurotransmitters, the production of normal neurons, and is implicated in the cardiovascular and the central nervous systems [31]. The B subtype of the receptor is known to be a biomarker in asthma and its agonists are used as therapeutic molecules as bronchoconstriction regulators [32,33]. Several studies associated SNPs in the *ADRA2B* gene with asthma and atopic diseases [34-37]. In the respiratory system, the activation of the adrenergic alpha-2A-receptor regulates the recovery of the cholinergic outflow to the airways in response to repeated allergen exposition and also leads to the release of proinflammatory cytokines [32,38]. This gene is also involved in the regulation of mood, response to stress, anxiety and autonomic function [39,40]. Consequently, interactions between physiological factors and emotions may modulate the severity and the symptoms of asthma at least partially through the *ADRA2A* gene.

The *ADH1B* gene is located on the long arm of the chromosome 4 and codes for the beta unit of class I alcohol dehydrogenase. This enzyme catalyzes the oxidation of alcohol to acetaldehyde [41]. SNPs in the alcohol dehydrogenase and the acetaldehyde dehydrogenase genes are involved in alcohol-induced hypersensitivity [42,43]. Moreover, the inhalation of acetaldehyde (alcohol) and a fast metabolism of ethanol can lead to a bronchoconstriction in asthmatic subjects [44]. Therefore, the *ADH1B* gene seems to be involved in an increase of airway obstruction in asthma by its effects on the alcohol metabolism.

## Conclusion

The association analysis on genes differently expressed in microarray studies of bronchial biopsies obtained from asthmatic and control subjects allowed confirming the association of four new genes to asthma and/or allergy. The known functions of these genes suggest that they could play a significant role in the pathogenesis of asthma. On the 100 genes identified as differently expressed in the Laprise *et al*. and Chamberland *et al.* studies, the combination of the four association studies performed by our team associated 9 genes after correction for multiple testing (9%). This study illustrates the potential of combining studies of expression and association in order to increase our understanding of the biology of complex traits, and more specifically to identify gene and biological pathways relevant to asthma.

## Competing interests

The authors declare that they have no competing interests.

## Authors' contributions

VTV performed the analysis and drafted the manuscript. MB performed a part of the analysis and contributed to the draft of the manuscript. ML contributed to the collection of the samples and to the design of the study for the microarrays. CL designed and directed the study as well as supervised and revised the manuscript. All authors read and approved the final manuscript.

## Supplementary Material

Additional file 1 **Table: S1 Acrobat reader (.pdf) http://get.adobe.com/fr/reader/.** Association before correction with asthma related phenotypes. This table lists all the associations before correction in the 85 studied genes for the three studied phenotypes, which are asthma, atopy and atopic asthma. The table also contains the number of tested SNPs, alleles and frequencies, Z scores and corrected threshold for each association with p≤0.05. Click here for file
