# Supplementary material for: From expression pattern to genetic association in asthma and asthma-related phenotypes
Source: BMC Res Notes. 2012 Nov 13;5:630. doi: 10.1186/1756-0500-5-630 (PMC3532380; doi:10.1186/1756-0500-5-630)
Supplement: Additional file 1 — Table: S1 Acrobat reader (.pdf) http://get.adobe.com/fr/reader/. Association before correction with asthma related phenotypes. This table lists all the associations before correction in the 85 studied genes for the three studied phenotypes, which are asthma, atopy and atopic asthma. The table also contains the number of tested SNPs, alleles and frequencies, Z scores and corrected threshold for each association with p≤0.05. [file 1756-0500-5-630-S1.pdf]

Additionnal table 1: Association before correction with asthma related phenotypes

Gene	Number of tested SNP	Corrected threshold	SNP	Allele	AF	Number of families	Z	p value		
								Asthma	Atopy	Atopic asthma
**Group 1: Immune signalling molecules**										
CD19	28	0.0025	4788114	T	7.3	55	-2.155	0.0312		
				C	92.7		2.155			
			9937676	G	37.7	110	-2.635			0.0084
				A	62.3		2.635			
			111506775	A	34.6	117	-1.920	0.0190		
				G	65.4		1.920			
			3922668	A	37.6	111	-2.697			0.0070
				G	62.4		2.697			
			8045276	T	26.5	89	1.974			0.0483
				A	73.5		-1.974			
**Group 2: Extracellular proteins**										
COL11A1	53	0.0011	1676486	A	20.6	107	2.192	0.0283		
				G	79.4		-2.192			
				A	20.6	87	2.091			0.0365
				G	79.4		-2.091			
			1241163	A	23.1	116	1.985	0.0471		
				C	76.9		-1.985			
			1085	A	23	115	2.059	0.0395		
				C	77		-2.059			
			1463039	T	6.8	44	2.712	0.0067		
				C	93.2		-2.712			
				T	6.8	43	-2.462			0.0138
				C	93.2		2.462			
			1415363	A	20.7	110	2.360	0.0183		
				G	79.3		-2.360			
				A	20.7	85	2.172			0.0299
				G	79.3		-2.172			
			7544816	A	5.3	35	-1.964	0.0495		
				C	94.7		1.964			
			10493986	C	27	121	2.031	0.0422		
				T	73		-2.031			
				C	27	97	2.127			0.0334
				T	73		-2.127			
			4338381	G	30.8	124	2.050	0.0404		
				A	69.2		-2.050			
			7543626	G	17.4	103	-2.376	0.0175		
				A	82.6		2.376			
				G	17.4	92	-1.960		0.0500	
				A	82.6		1.960			

Gene	Number of tested SNP	Corrected threshold	SNP	Allele	AF	Number of families	Z	p value		
								Asthma	Atopy	Atopic asthma
MUC2	13	0.0030	7103978	G	7.1	39	-1.987			0.0469
				A	92.9		1.987			
COL2A1	24	0.0020	4760607	G	10.4	44	2.168	0.0302		
				T	89.6		-2.168			
			917055	A	15.1	95	2.072	0.0382		
				G	84.9		-2.072			
			1034762	A	16.9	105	2.164	0.0300		
				C	83.1		-2.164			
			6580647	A	19.1	110	2.724	0.0064		
				C	80.9		-2.724			
			7305954	C	19.2	111	2.649	0.0081		
				T	80.8		-2.649			
AGC1	32	0.0012	12439075	T	33.1	121	2.161	0.0307		
				C	66.9		-2.161			
			11073813	G	33.3	120	2.108	0.0350		
				A	66.7		2.108			
			11630178	C	33.5	99	2.695	0.0070		
				T	66.5		-2.695			
				C	33.5	82	3.589		0.0003	
				T	66.5		-3.589			
				C	33.5	85	3.900			**0.0001**
				T	66.5		-3.900			
			938605	G	17.6	101	-2.203	0.0276		
				A	82.4		2.203			
				G	17.6	84	-2.086			0.0370
				A	82.4		2.086			
			11632435	G	12.9	75	-2.254		0.0242	
				A	87.1		2.254			
			8031532	G	16.8	82	-2.458		0.0140	
				T	83.2		2.458			
			12905452	A	27.9	95	2.635		0.0084	
				C	72.1		-2.635			
				A	27.9	100	2.745			0.0061
				C	72.1		-2.745			
			7180356	T	29.9	95	-2.166		0.0303	
				C	70.1		2.166			
				T	29.9	100	2.134			0.0328
				C	70.1		-2.134			
			4932432	C	31.6	106	2.095		0.0361	
				A	68.4		-2.095			
				C	31.6	197	2.690			0.0071
				A	68.4		-2.690			

Gene	Number of tested SNP	Corrected threshold	SNP	Allele	AF	Number of families	Z	p value		
								Asthma	Atopy	Atopic asthma
AGC1	32	0.0012	938613	A	33.5	109	2.154		0.0312	
				G	66.5		-2.154			
				A	33.5	110	2.570			0.0102
				G	66.5		-2.570			
FBLN1	30	0.0011	3810631	T	36.4	134	-1.962	0.0498		
				C	63.6		1.962			
			5765475	C	42.5	115	2.144			0.0321
				T	57.5		-2.144			
			2051616	G	40.6	107	2.182	0.0291		
				A	59.4		-2.182			
**Group 3: Immune response**										
HLA-DPB1	24	0.0028	1431403	C	35.6	144	-2.043	0.0411		
				T	64.4		2.043			
			9277357	G	36.2	138	-2.609	0.0091		
				A	63.8		2.609			
				G	36.2	111	-2.500			0.0124
				A	63.8		2.500			
			9277396	A	37.7	143	-2.680	0.0074		
				G	62.3		2.680			
				A	37.7	124	-2.199		0.0279	
				G	62.3		2.199			
				A	37.7	114	-2.733			0.0063
				G	62.3		2.733			
			9277464	T	34.9	139	-2.530	0.0114		
				C	65.1		2.530			
				T	34.9	119	-2.014		0.0440	
				C	65.1		2.014			
				T	34.9	111	-2.553			0.0107
				C	65.1		2.553			
			9277535	G	34.9	138	-2.591	0.0096		
				A	65.1		2.591			
				G	34.9	118	-2.083		0.0372	
				A	65.1		2.083			
				G	34.9	110	-2.628			0.0086
				A	65.1		2.268			
			9277554	T	37.6	142	-2.869	0.0041		
				C	62.4		2.869			
				T	37.6	125	-2.256		0.0241	
				C	62.4		2.256			
				T	37.6	114	-2.649			0.0081
				C	62.4		2.649			

Gene	Number of tested SNP	Corrected threshold	SNP	Allele	AF	Number of families	Z	p value		
								Asthma	Atopy	Atopic asthma
HLA-DPB1	24	0.0028	9277555	A	30.5	128	-2.501	0.0124		
				G	69.5		2.501			
				A	30.5	113	-2.539		0.0111	
				G	69.5		2.539			
				A	30.5	102	-2.660			0.0078
				G	69.5		2.660			
			9277565	T	27.8	124	-2.588	0.0096		
				C	72.2		2.588			
				T	27.8	108	-2.433		0.0150	
				C	72.2		2.433			
				T	27.8	99	-2.673			0.0075
				C	72.2		2.673			
			10484569	A	9.7	65	-2.225	0.0261		
				G	90.3		2.225			
				A	9.7	59	-2.714		0.0067	
				G	90.3		2.714			
				A	9.7	52	-2.390			0.0168
				G	90.3		2.390			
			3128917	G	30.5	128	-2.501	0.0124		
				T	69.5		2.501			
				G	30.5	113	-2.539		0.0111	
				T	69.5		2.539			
				G	30.5	102	-2.660			0.0078
				T	69.5		2.660			
			2281388	A	6.4	46	-2.255		0.0241	
				G	93.6		2.255			
			3117222	T	30.5	127	-2.400	0.0164		
				C	69.5		2.400			
				T	30.5	112	-2.427		0.0152	
				C	69.5		2.427			
				T	30.5	102	-2.660			0.0078
				C	69.5		2.660			
			3117221	T	27.8	124	-2.468	0.0136		
				C	72.2		2.468			
				T	27.8	107	-2.369		0.0178	
				C	72.2		2.369			
				T	27.8	99	-2.619			0.0088
				C	72.2		2.619			
			6914651	G	31.6	111	-2.305	0.0212		
				A	68.4		2.305			
				G	31.6	102	-2.955		0.0031	
				A	68.4		2.955			
				G	31.6	92	-2.863			0.0042
				A	68.4		2.863			

Gene	Number of tested SNP	Corrected threshold	SNP	Allele	AF	Number of families	Z	p value		
								Asthma	Atopy	Atopic asthma
MS4A1	27	0.0037	11230335	A	8.9	60	2.828	0.0047		
				C	91.1		-2.282			
CEACAM5	9	0.0050	7257798	C	23	117	2.042	0.0412		
				A	77		-2.042			
IGHG1	39		2040459	A	11.8	73	2.034	0.0420		
				C	88.2		-2.034			
		0.0028	2105992	A	18.4	96	2.334	0.0196		
				G	81.6		-2.334			
				A	18.4	81	1.993		0.0463	
				G	81.6		-1.993			
				A	18.4	79	2.433			0.0150
				G	81.6		-2.433			
			17112712	A	29.8	94	2.440		0.0147	
				G	70.2		-2.440			
				A	29.8	95	2.219			0.0265
				G	70.2		-2.219			
			1544317	G	30.4	99	2.376		0.0175	
				A	69.6		-2.376			
				G	30.4	98	2.134			0.0328
				A	69.6		-2.134			
IGL@	74	0.0012	5760189	T	27.2	114	2.194	0.0282		
				C	72.8		-2.194			
				T	27.2	103	2.204		0.0275	
				C	72.8		-2.204			
				T	27.2	96	2.691			0.0071
				C	72.8		-2.691			
			2001106	C	8.8	49	-2.224		0.0262	
				T	91.2		2.224			
				C	8.8		-2.066			0.0389
				T	91.2		2.066			
			3788368	C	10.4	58	-2.061		0.0393	
				T	89.6		2.061			

Gene	Number of tested SNP	Corrected threshold	SNP	Allele	AF	Number of families	Z	p value		
								Asthma	Atopy	Atopic asthma
**Group 4: Intracellular signalling**										
PTPRC	35	0.0012	1052238	T	47.9	135	-2.230	0.0257		
				C	52.1		2.230			
				T	47.9	117	-2.384		0.0171	
				C	52.1		2.384			
				T	47.9	115	-2.333			0.0196
				C	52.1		2.333			
			7522020	T	34.3	131	2.055	0.0399		
				G	65.7		-2.055			
				T	34.3	104	2.085		0.0371	
				G	65.7		-2.085			
				T	34.3	107	2.137			0.0326
				G	65.7		-2.137			
			9803750	T	32	118	-2.863	0.0042		
				C	68		2.863			
				T	32	108	-3.034		0.0024	
				C	68		3.034			
				T	32	99	-2.880			0.0040
				C	68		2.880			
			2359952	G	32.6	117	-2.664	0.0077		
				A	67.4		2.664			
				G	32.6	106	-2.427		0.0151	
				A	67.4		2.427			
				G	32.6	98	-2.710			0.0067
				A	67.4		2.710			
			3754098	T	8.4	51	-2.502			0.0123
				C	91.6		2.502			
			1932436	G	28.1	131	-2.277	0.0228		
				A	71.9		2.277			
			14575169	A	34.4	107	-2.064		0.0390	
				C	65.6		2.064			
			10919584	A	33.6	107	-2.115		0.0344	
				G	66.4		2.115			
G1P3	3	0.0250	1247653	G	43.4	133	-2.353	**0.0186**		
				A	56.6		2.353			
SYK	56	0.0006	169724	C	9	57	-2.043		0.0411	
				T	91		2.043			
			10993705	T	7.3	103	2.086		0.0370	
				G	92.7		-2.086			
**Group 5: Proteolytic enzymes**										
CTSC	18	0.0028	217111	G	49.9	108	2.084			0.0372
				A	50.1		-2.084			
PSMA6	5	0.0063	10141237	G	9.3	62	-1.975	0.0482		
				A	90.7		1.975			

Gene	Number of tested SNP	Corrected threshold	SNP	Allele	AF	Number of families	Z	p value		
								Asthma	Atopy	Atopic asthma
SPINK5	22	0.0023	2287770	G	6.6	41	2.321		0.0203	
				A	93.4		-2.321			
			2287769	T	6.6	41	2.321		0.0203	
				C	93.4		-2.321			
			6580523	A	87.3	84	-2.169	0.0301		
				C	12.7		2.169			
**Group 6: Transmembrane proteins**										
KLRC3	4	0.0127	2734565	C	25	125	1.975	0.0482		
				T	75		-1.975			
				C	25	109	2.166		0.0303	
				T	75		-2.166			
			2617170	T	25	125	1.975	0.0482		
				C	75		-1.975			
				T	25	108	2.183		0.0290	
				C	75		-2.183			
ADRA2A	1	0.0250	11195299	A	6.3	56	-2.588	**0.0097**		
				G	93.7		2.588			
CLCA2	13	0.0034	4912463	A	25.8	114	2.405		0.0162	
				C	74.2		-2.504			
				A	25.8	108	2.801			0.0051
				C	74.2		-2.801			
KCNJ16	21	0.0017	1990197	G	46.4	139	2.019	0.0435		
				A	53.6		-2.019			
				G	46.4	117	2.078		0.0377	
				A	53.6		-2.078			
			12943869	G	31.1	110	2.018		0.0436	
				A	68.9		-2.018			
**Group 7: Free radical metabolism**										
CYBA	5	0.0054	899729	A	50	126	-1.990		0.0466	
				C	50		1.990			
**Group 8: Gene transcription**										
ZNF38	4	0.0085	11550034	G	18.1	102	-1.986	0.0470		
				A	81.9		1.986			
**Group 10: Cell adhesion molecules**										
ITGB2	33	0.0012	2070947	G	34.5	129	-2.031	0.0423		
				A	65.5		2.031			
				G	34.5	108	-2.050			0.0404
				A	65.5		2.050			

Gene	Number of tested SNP	Corrected threshold	SNP	Allele	AF	Number of families	Z	p value		
								Asthma	Atopy	Atopic asthma
**Group 11: Complement components**										
C7	35	0.0014	13175903	G	24.1	123	-2.745	0.0060		
				A	75.9		2.745			
				G	24.1	98	-2.204		0.0275	
				A	75.9		2.204			
				G	24.1	97	-2.407			0.0161
				A	75.9		2.407			
			16870514	A	9.6	53	1.995			0.0461
				G	90.4		-1.995			
**Group 12: Metabolic enzymes**										
ALDH3A1	13	0.0078	1989379	A	25	129	-1.965	0.0494		
				G	75		1.965			
			2228100	C	23.1	112	2.032	0.0422		
				G	76.9		-2.032			
FA2H	14	0.0024	12919626	A	11.6	84	-2.279	0.0226		
				G	88.4		2.279			
				A	11.6	68	-2.730			0.0063
				G	88.4		2.730			
			49999690	A	36.6	109	2.155		0.0312	
				G	63.4		-2.155			
ADH1B	15	0.0030	1826909	C	31.3	133	2.807	0.0050		
				T	68.7		-2.807			
				C	31.3	114	2.400		0.0164	
				T	68.7		-2.400			
				C	31.3	112	2.356			0.0185
				T	68.7		-2.356			
			13103321	T	47.7	135	-3.040	**0.0024**		
				G	52.3		3.040			
				T	47.7	114	-2.566		0.0103	
				G	52.3		2.566			
				T	47.7	114	-2.621			0.0088
				G	52.3		2.621			
			1353621	C	47.3	136	-2.848	0.0044		
				T	52.7		2.848			
				C	47.3	116	-2.436		0.0149	
				T	52.7		2.436			
				C	47.3	115	-2.403			0.0063
				T	52.7		2.403			
			1159918	A	32.3	126	2.329	0.0199		
				C	67.7		-2.329			
				A	32.3	106	2.194		0.0282	
				C	67.7		-2.194			

Gene	Number of tested SNP	Corrected threshold	SNP	Allele	AF	Number of families	Z	p value		
								Asthma	Atopy	Atopic asthma
PIK3R1	4	0.0065	34306	A	31.3	82	-2.154		0.0313	
				G	68.7		2.154			
				A	31.3	81	-2.021			0.0433
				G	68.7		2.021			
**Group 13: Structural proteins**										
TENASCIN C	36	0.0012	1330368	G	45.3	134	2.019		0.0435	
				A	54.7		-2.019			
			1060545	C	45.1	132	2.020		0.0434	
				A	54.9		-2.020			
			1757095	T	8.5	48	2.157		0.0310	
				C	91.5		-2.157			

Abbreviations : SNP = Single Nucletodie Polymorphism, AF= Minor Allele Frequencie, Z= Z score
